# Distribution of GnRH and Kisspeptin Immunoreactivity in the Female Llama Hypothalamus

**DOI:** 10.3389/fvets.2020.597921

**Published:** 2021-02-02

**Authors:** Marco Berland, Luis Paiva, Lig Alondra Santander, Marcelo Héctor Ratto

**Affiliations:** ^1^Departamento de Ciencias Veterinarias y Salud Pública, Facultad de Recursos Naturales, Universidad Católica de Temuco, Temuco, Chile; ^2^Instituto de Ciencia Animal, Facultad de Ciencias Veterinarias, Universidad Austral de Chile, Valdivia, Chile

**Keywords:** LHRH, metastin, OVLT, median eminence, induced ovulation, llama glama, camelids

## Abstract

Llamas are induced non-reflex ovulators, which ovulate in response to the hormonal stimulus of the male protein beta-nerve growth factor (β-NGF) that is present in the seminal plasma; this response is dependent on the preovulatory gonadotrophin-releasing hormone (GnRH) release from the hypothalamus. GnRH neurones are vital for reproduction, as these provide the input that controls the release of luteinizing hormone (LH) and follicle-stimulating hormone (FSH) from the pituitary gland. However, in spontaneous ovulators, the activity of GnRH cells is regulated by kisspeptin neurones that relay the oestrogen signal arising from the periphery. Here, we investigated the organisation of GnRH and kisspeptin systems in the hypothalamus of receptive adult female llamas. We found that GnRH cells exhibiting different shapes were distributed throughout the ventral forebrain and some of these were located in proximity to blood vessels; sections of the mediobasal hypothalamus (MBH) displayed the highest number of cells. GnRH fibres were observed in both the organum vasculosum laminae terminalis (OVLT) and median eminence (ME). We also detected abundant kisspeptin fibres in the MBH and ME; kisspeptin cells were found in the arcuate nucleus (ARC), but not in rostral areas of the hypothalamus. Quantitative analysis of GnRH and kisspeptin fibres in the ME revealed a higher innervation density of kisspeptin than of GnRH fibres. The physiological significance of the anatomical findings reported here for the ovulatory mechanism in llamas is still to be determined.

## Introduction

In female mammals, ovulation relies on the integration of different central and peripheral components that establish reciprocal interactions. At the pituitary level, the preovulatory release of the luteinizing hormone (LH) from gonadotroph cells is led by the gonadotrophin-releasing hormone (GnRH) released from the hypothalamus (1).

GnRH neurones originate in the olfactory placode in the early embryonic life and migrate through the vomeronasal axons to the basal forebrain during development, stopping their migratory journey ‘randomly’ along the basal forebrain (2). In the adult hypothalamus, GnRH cells are distributed in a bilateral long scattered continuum (2, 3) that exhibit distinct species-related number and distribution. The preoptic area (POA)–anterior hypothalamus and the mediobasal hypothalamus (MBH) are two regions known to harvest large numbers of GnRH somas (4), but GnRH somas and fibres can also be found in other brain areas, such as the olfactory bulb and hippocampus (5). Approximately 50% of the GnRH cells send their fibres to the median eminence (ME) where nerve endings release their products into the hypophyseal portal system that transport GnRH molecules to the pituitary (6, 7). The activity of these hypophysiotropic GnRH cells is regulated by several inputs and factors (such as environmental cues) that vary in different species (8).

Kisspeptin neurones (encoding the gene *KISS1*) are recognised as the main input involved in the activation of GnRH cells that lead to the preovulatory GnRH/LH surge by relaying oestrogen signalling in spontaneous ovulators (9, 10). In seasonal breeders, kisspeptin cells have also been shown to be modulated by photoperiodic changes, and so they are directly involved in the control of reproductive seasonality in different species (10, 11). Two populations of kisspeptin cells located in the POA (or homologous areas) and the arcuate nucleus (ARC)–co-expressing neurokinin B and dynorphin in the latter, and so-called KNDy neurones–of the hypothalamus can be found in several species (12). So, when oestrogen concentrations rise, *KISS1* expression is upregulated in POA cells, and they become activated, prompting the activation of GnRH cells and their product release (13, 14). Evidence indicates that kisspeptin may act directly over GnRH somas (15, 16), but also, perhaps, at the ME in some species, including rats (17), sheep (18), and humans (19).

The physiological role of kisspeptin in induced ovulation species is not fully understood. In the musk shrew (*Suncus murinus*), *KISS1* expression seems to be like in spontaneous ovulators, but mating is reported as the critical cue for the activation (by means of c-Fos expression) of POA *KISS1* cells (20), indicating kisspeptin cells as a potential player involved in the ovulatory mechanism. Llamas are also induced ovulators, however, in this species, ovulation occurs in response to the hormonal [but not reflex (21)] stimulus of the protein beta-nerve growth factor (β-NGF) that is present in the male seminal plasma (22, 23); the resulting LH surge and ovulation have been shown to be dependent on GnRH release (24), implying the participation of a central mechanism. Although the involvement of kisspeptin in this response has been suggested (25), the organisation of kisspeptin systems and its physiological role is not clear.

In this study, we characterise the distribution of GnRH and kisspeptin cells and their fibres (i.e., axons and dendrites) in the hypothalamus of receptive female llamas using immunohistochemistry. We also carried out dual immunofluorescence in sections containing the ME to analyse the density of GnRH- and kisspeptin-containing fibres, which are next to fenestrated blood vessels of the hypophyseal portal system.

## Methods

### Animals

Llamas were maintained in the llama research farm of the Universidad Austral de Chile, Valdivia, Chile (39°38′S, 73°5′W, and 19 m above sea level) and were given *ad libitum* access to water and pastures supplemented with hay and feed pellets (14% crude protein, 2.5% crude fat, 12% crude fibre); female llamas were maintained separately from males. Three (3) adult non-pregnant, non-lactating female llamas, weighing 120–140 kg (4–8 years old), were used in this study. These llamas were daily examined by transrectal ultrasonography using a 7.5 MHz transducer coupled with an Aloka SSD-500 scanner (Aloka Co., Ltd., Tokyo, Japan) to determine follicular growth; females exhibiting a follicle ≥8 mm in diameter that grew for 3 consecutive days were considered sexually receptive and euthanised. Experiments were conducted in June.

All the procedures were carried out in accordance with the Chilean Animal Protection Act (No. 20380; 2009) and the regulations and approved procedures (ref. 253/2015) by the University Bioethical Committee.

### Tissue Collection and Immunohistochemistry

Llamas were terminally anesthetised by an injection of sodium pentobarbital (80 mg/kg i.v.), and then the jugular veins were cannulated for exsanguination. Once death was confirmed, the brain was quickly removed and dissected, and the hypothalamic chunk was fixed by immersion in 4% paraformaldehyde in phosphate-buffered saline (PBS) for 72 h. The tissue was embedded in paraffin and then cut using a rotary microtome to obtain 5-μm serial sections; two hypothalami were cut coronally, and one hypothalamus was cut sagittally. One in every 30 sequentially cut sections (i.e., 150 μm) was mounted onto slides.

Conventional immunohistochemistry methods were used. Sections were washed in 0.02 M potassium phosphate-buffered saline (KPBS; pH 7.4) between steps. In brief, paraffin-embedded sections were dewaxed, and heat-induced epitope retrieval was performed for 20 min using 1× Tris–EDTA buffer pH 9.0 at 90°C. Then, the endogenous peroxidase activity was blocked by incubating the sections in 2% H_2_O_2_ in cold methanol for 20 min. To prevent the background staining caused by interactions of antibodies with the tissue, sections were incubated in blocking buffer solution containing 0.02 M KPBS + 0.5% Triton X-100 + 2% normal goat serum for 30 min. Sections were then incubated overnight at 4°C in blocking buffer containing either mouse monoclonal HU11B anti-GnRH-I antibody (cat. sc-32292; Santa Cruz Biotechnology Inc., Dallas, TX, USA) diluted at 1:1,000 or rabbit polyclonal anti-kisspeptin antibody (cat. AB9754; Millipore, Billerica, MA, USA) diluted at 1:1,000. Following this, sections were washed and incubated for 1 h at room temperature with secondary goat anti-mouse IgG or goat anti-rabbit IgG biotinylated antibodies (cat. 115-065-003 and 111-065-003, respectively; Jackson ImmunoResearch Inc., West Grove, PA, USA) diluted at 1:500 in 0.02 M KPBS and then incubated in Vectastain Elite ABC Kit (cat. PK-6100; Vector Laboratories Inc., Burlingame, CA, USA) following the manufacturer's instructions. The immunoreaction was revealed by a solution containing 0.05% diaminobenzidine + 0.015% H_2_O_2_ in 0.02 M KPBS. Then, sections were counterstained with haematoxylin, dehydrated in increasing concentrations of ethanol and coverslipped.

For double immunofluorescence, sections were treated and incubated simultaneously with primary antibodies as described above, but the immunoreactions were visualised by incubating the sections with donkey anti-mouse IgG Alexa Fluor 594 and donkey anti-rabbit IgG Alexa Fluor 488 (cat. A-21203 and A-21206, respectively; Invitrogen, Carlsbad, CA, USA) diluted at 1:500 in blocking buffer containing 2% donkey normal serum for 1 h at room temperature. Sections were counterstained using 4', 6-diamidino-2-phenylindole (DAPI).

**Figure 1:**
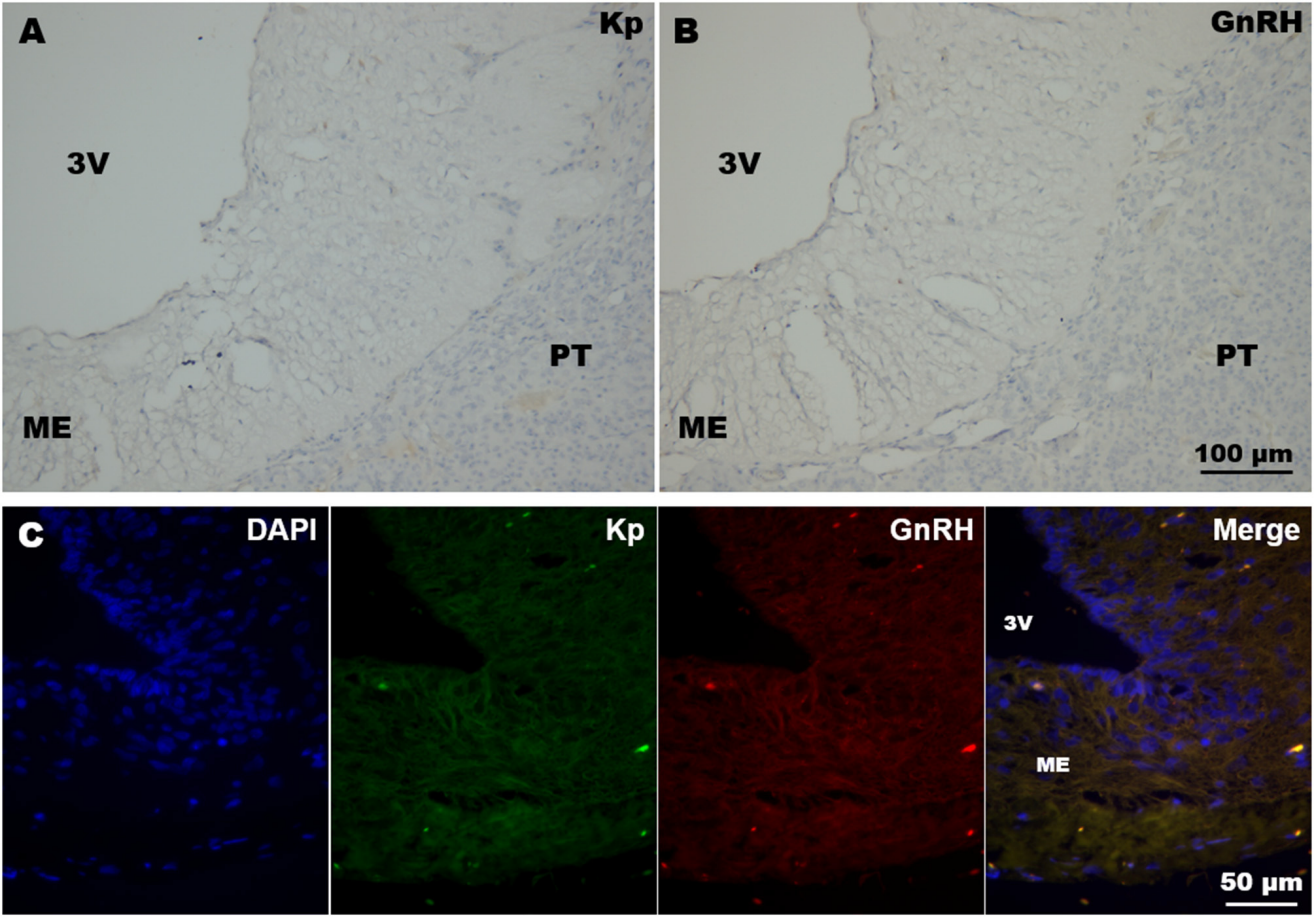
Immunohistochemistry negative controls. **(A,B)** Coronal sections of llama ME showing DAB immunohistochemistry where **(A)** kisspeptin and **(B)** GnRH primary antibodies were omitted. **(C)** Example of double immunofluorescence in llama ME (coronal section) incubated without kisspeptin (Kp) and GnRH primary antibodies. 3v, third ventricle; ME, median eminence; PT, pituitary.

In all immunohistochemistry and immunofluorescence experiments, the specificity of immunoreactions was tested by omission of primary antibodies in control sections containing the ME; no immunoreaction was detected in these sections (Figure 1).

### Imaging and Data Analysis

Images were captured using a Nikon digital camera attached to an upright Nikon Eclipse i80 microscope (Nikon Instruments Inc., Tokyo, Japan). Since thin sections make it difficult to identify cell shapes, only clearly distinguishable cell somas exhibiting a single or more processes parallel to the coronal plane were used for description and illustration purposes. Dendrites and axons are irrespectively reported as fibres similarly as Witkin et al. (26). For quantification of GnRH and kisspeptin fibres in the ME, four sections per animal were analysed; in each of these sections, four digital images of non-overlapping microscopic fields at 40× magnification were captured at different sectors of the ME. The area (μm^2^) covered by GnRH and kisspeptin fibres in each image was calculated using a PC running Fiji version 1.52p; briefly, red (i.e., GnRH) and green (i.e., kisspeptin) channels of image files were separately converted into 8-bit black and white images, thresholded using the same values, and the areas calculated using the Analyze Particle macro. The area calculated in each image was averaged for each section; the values in each section were averaged in each animal. The fibre density was calculated as the percentage of immunoreactive material covering the whole image area (234 × 292 μm^2^). Values are expressed as the mean + SEM; only descriptive statistics were used in this study.

## Results

### Kisspeptin

**Figure 2:**
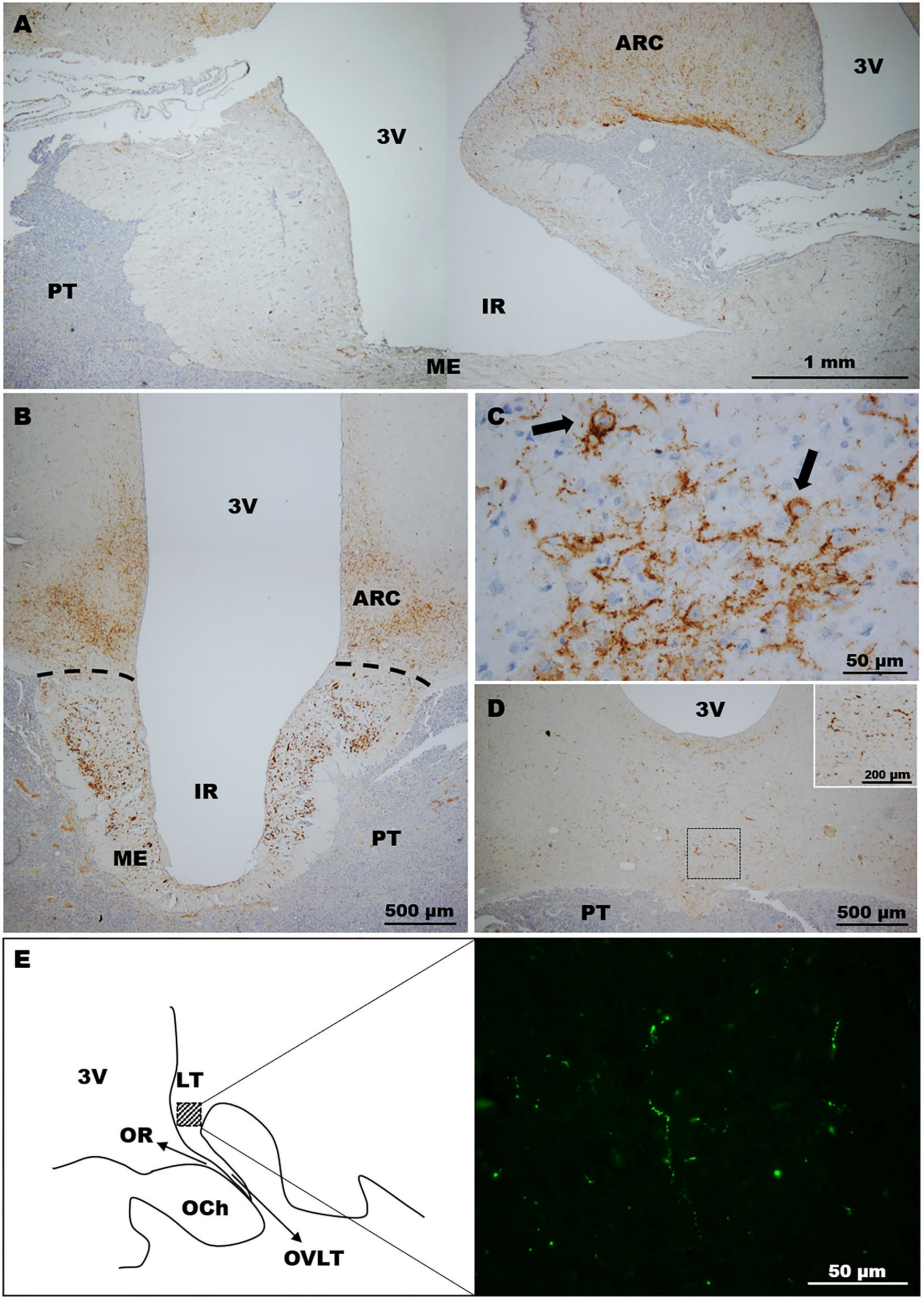
Distribution of kisspeptin in the llama hypothalamus. **(A)** Sagittal image showing the distribution of kisspeptin fibres in the MBH. **(B)** Coronal section. Immunolabelled fibres were located mostly in the ARC and ME. **(C)** Image of the ARC showing (arrows) the labelling of kisspeptin somas. **(D,E)** Kisspeptin fibres were moderately found in some areas of the **(D)** anterior hypothalamus and **(E)** POA. **(E)** Left panel shows a schematic representation of the POA on a sagittal section indicating in the square area the high magnification picture (right panel) displaying kisspeptin immunolabelled fibres at this zone. 3v, third ventricle; ARC, arcuate nucleus; IR, infundibular recess; LT, laminae terminalis; ME, median eminence; OCh, optic chiasm; OR, optic recess; OVLT, organum vasculosum laminae terminalis; PT, pituitary.

In llamas, immunohistochemistry revealed the abundant presence of varicose fibres exhibiting different calibres in the ARC region, and also the internal zone of ME (Figures 2A,B). In the latter, the abundant presence of kisspeptin-immunoreactive material was found in the lateral walls of the ME. Analysis of the ARC showed the presence of some somas (Figure 2C).

Moderate kisspeptin fibres were found in the ventral region of the anterior hypothalamus (Figure 2D). Sagittal sections revealed the presence of fibres in the POA (lamina terminalis; Figure 2E). Somas were not detected in sections containing the POA and anterior hypothalamus in the llamas analysed.

### GnRH

**Figure 3:**
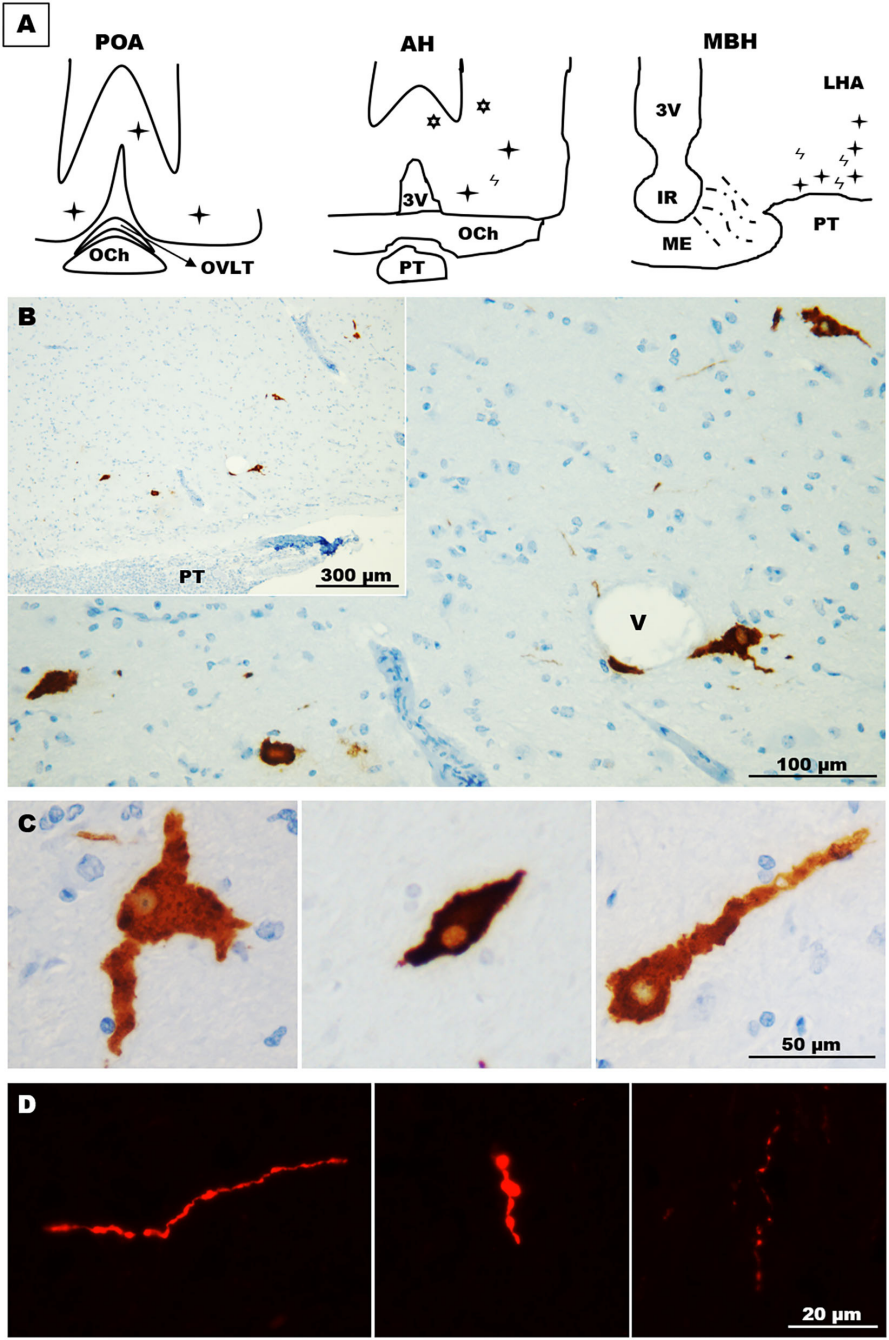
Distribution and morphology of GnRH cells in the llama hypothalamus. **(A)** Schematic representation of the relative abundance of GnRH cells in the POA, anterior hypothalamus, and MBH. 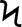 = fibres, 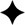 = hypothalamic cells, 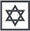 = extrahypothalamic cells. **(B)** Coronal section showing scattered GnRH cells in the ventro-lateral area of the MBH. **(C)** GnRH cells exhibited different forms, including multipolar (left panel), fusiform (middle panel) and unipolar (right panel) shapes. **(D)** Immunofluorescences showing the different shapes and calibres of GnRH fibres found in the llama hypothalamus. 3v, third ventricle; AH, anterior hypothalamus; IR, infundibular recess; LHA, lateral hypothalamic area; MBH, mediobasal hypothalamus; ME, median eminence; OCh, optic chiasm; OVLT, organum vasculosum laminae terminalis; POA, preoptic area; PT, pituitary; V, blood vessel.

Immunohistochemistry revealed GnRH somas located in different areas of the llama hypothalamus. Two (2) to 3 GnRH somas per section were found in sections containing the POA, which were mainly found in the diagonal band of Broca. In the anterior hypothalamus, somas (~1–2) were found in the latero-ventral area, but also in extrahypothalamic sites near to the lateral ventricles (Figure 3A). Sections of the MBH exhibited the greatest number of GnRH somas, where 3–5 GnRH cells per section were detected in the latero-ventral area of the hypothalamus (Figures 3A,B); in some sections, GnRH cells were in close contact to blood vessels (Figure 3B). These neurones displayed different forms, including multipolar, bipolar, and monopolar as shown in Figure 3C. In most sections, different neural fibres were detected in the hypothalamic parenchyma, which exhibited different lengths and variable calibres (Figure 3D).

**Figure 4:**
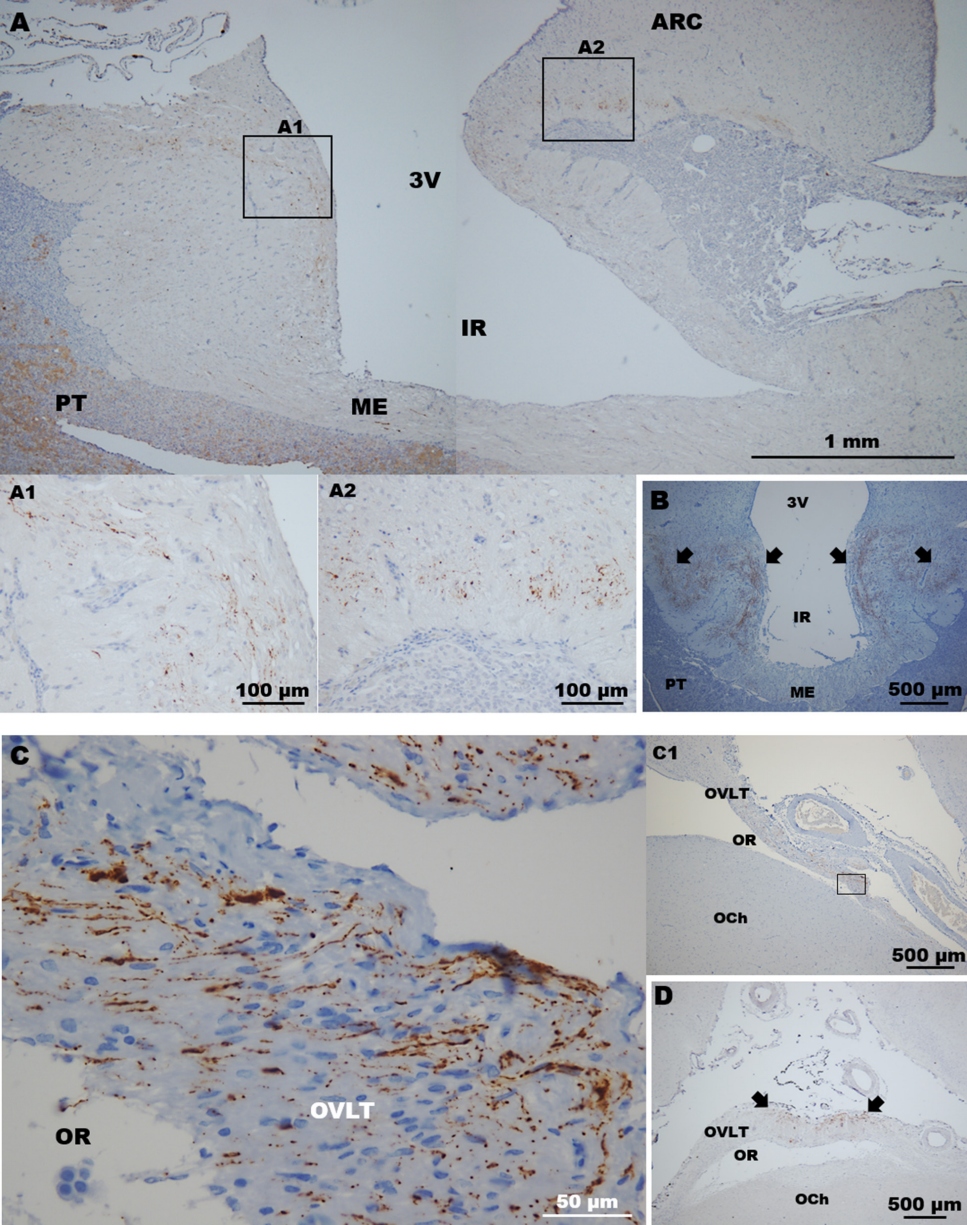
Detection of GnRH fibres in circumventricular organs. **(A)** In sagittal sections, GnRH fibres were found in the **(A1)** rostral (pre-infundibular) and **(A2)** caudal (post-infundibular) areas of the ME. **(B)** Coronal section showing GnRH fibres in the lateral walls (arrows) of the ME surrounding the infundibulum; virtually none fibres were detected in the ventral wall of the ME. **(C)** High magnification image of **(C1)** a sagittal section showing GnRH fibres in the ventral area (square) of the OVLT. **(D)** Coronal section. Most of the fibres were found in the medial area (arrows) of the OVLT. 3v, third ventricle; ARC, arcuate nucleus; IR, infundibular recess; ME, median eminence; OCh, optic chiasm; OR, optic recess; OVLT, organum vasculosum laminae terminalis; PT, pituitary.

In the ME, immunolabelling of GnRH showed the moderate presence of GnRH fibres that were mainly located in the pre-infundibular and post-infundibular regions as shown in Figure 4A. Fibres were also found in the rostral and caudal walls of the ME, but they were rarely detected in the ventral wall of the ME. Fibres were detected in the internal zone of the ME, and some of these penetrated into the ME external zone; analysis of coronal sections revealed that most fibres were mainly located in the lateral walls of the ME (Figure 4B).

In the organum vasculosum laminae terminalis (OVLT), the presence of GnRH fibres varied in each section analysed; some of these displayed the abundant presence of fibres mainly located in the ventral OVLT, next to the narrowest area of the optic recess as presented in Figure 4C; coronal sections revealed that most of the fibres were located in the medial area of the OVLT (Figure 4D). These fibres exhibited different shapes, including long punctuate axon-like fibres, and thick processes (Figure 4C).

### Kisspeptin and GnRH Fibre Relationship in the ME

**Figure 5:**
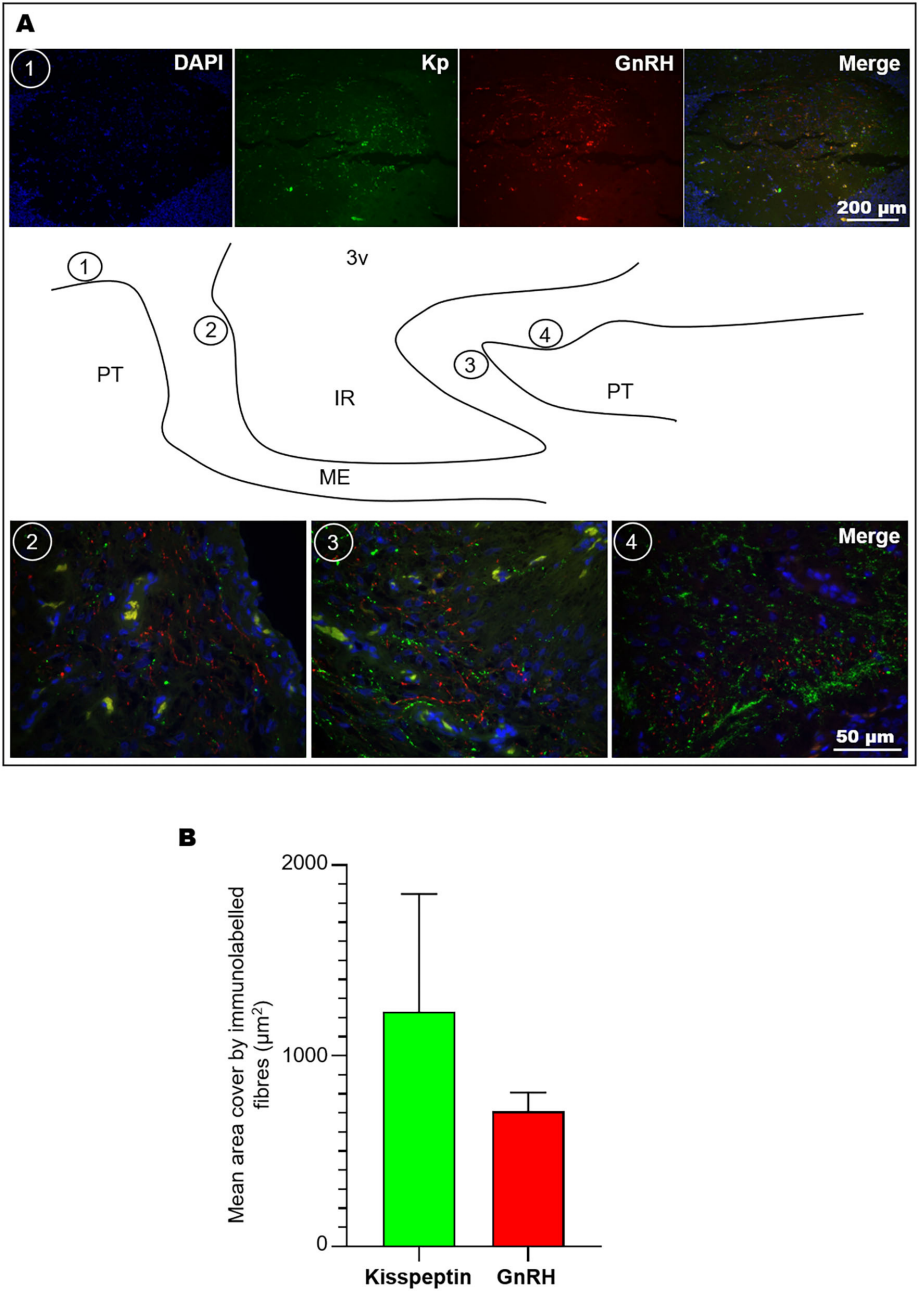
Co-labelling of kisspeptin and GnRH fibres in the llama ME. **(A)** Coronal (1) and sagittal images (2, 3, 4) showing the abundance of kisspeptin (Kp) and GnRH fibres at different levels of the ME as indicated in the sagittal schematic representation; scarce fibres were found in the ventral walls of the ME. **(B)** Mean (+SEM) area covered by immunolabelled kisspeptin and GnRH fibres in 40× microphotographs. 3v, third ventricle; IR, infundibular recess; ME, median eminence; PT, pituitary.

The ME plays a pivotal role through the release of hypophysiotropic factors, including GnRH, which stimulates the preovulatory LH release from the pituitary gland. To determine the configuration of this site in receptive llamas, double immunofluorescence against kisspeptin and GnRH was conducted, showing that GnRH and kisspeptin fibres were closely distributed in the ME, but no obvious contacts between these fibres were observed. Analysis of high magnification images of different sectors of the ME (Figure 5A) showed a greater amount of kisspeptin than of GnRH immunoreactive material (2:1 ratio). Kisspeptin fibres covered an average area of 1,230.9 ± 618.8 vs. 708.9 ± 98.8 μm of GnRH fibres (Figure 5B); these values represent 1.8 ± 0.9 and 1.0 ± 0.2% of the total area (40× image) analysed, respectively.

## Discussion

In this study, we investigated the presence and distribution of kisspeptin and GnRH cells in the adult female llama hypothalamus; two crucial neuroendocrine systems involved in the LH surge and ovulation in mammals. We found that kisspeptin is abundantly expressed in the llama MBH, where kisspeptin cells were detected in the ARC similarly to that reported in other spontaneous and induced ovulators. We also found GnRH cells and fibres distributed throughout rostral and caudal regions of the llama hypothalamus.

GnRH cells originate in the olfactory placode and migrate through the basal forebrain stopping their migration in a ‘random’ manner, and so the somas display a scattered distribution in the vertebrate adult hypothalamus (27). Our results show that the llama GnRH cells distribute unevenly throughout the hypothalamus similarly as described in other induced (28–30) and also spontaneous ovulators (26, 31, 32). Although the number of GnRH cells was not quantified in this study because of the use of thin sections sampled every 150 μm, sections of MBH contained approximately twice the number of cells than rostral sections containing the POA–anterior hypothalamus. This result is consistent with Carrasco et al. (33), who reported a higher number of cells in the MBH than in the POA, showing a proportion of 2:1.

In the present study, we used the HU11B monoclonal antibody that is reported (34) to exhibit high-specificity for sequential, rather than conformational, GnRH decapeptide (cleaved) structure, and which is able to bind GnRH fragments comprising up to four NH_2_-terminal amino acid residues. Furthermore, Urbanski (34) reports virtually no immunoactivity against salmon and chicken GnRH-I variants in which two and one amino acid residues of this fragment are exchanged, respectively. This implies that the cells labelled here are unlikely to include cells expressing other molecular GnRH forms than the encoded by the ‘mammalian’ gene (*GNRH1*) that is highly conserved among mammalian species except in guinea pig (35). Although originally identified in chicken, GnRH-II expressing cells have been found in the brain of macaques and humans, and also in the induced ovulator musk shrew (36). The GnRH-II peptide shares 70% of homology with the mammalian GnRH-I as the amino acid residues of positions 5, 7, and 8 next to the NH_2_-terminus are substituted (35, 37). Whether this GnRH form coexists in the llama brain has not been determined so far.

Kisspeptin cells of the POA and ARC participate in the modulation of the activity of GnRH cells; the former population has been linked to the control of the preovulatory GnRH surge in spontaneous ovulators. Here, we fail to detect the presence of POA kisspeptin cells in llamas; it is not clear whether this result was a consequence of a lack of immunoreactivity of this population or the kisspeptin system displays a different distribution in this species, similarly as reported in mares (38). Recently, it has been reported the presence of a low number of kisspeptin cells in the POA–anterior hypothalamus regions; this number was only an eighth of the kisspeptin ARC cells detected in adult llamas exhibiting a preovulatory follicle (39). Although the discrepancy between these results is apparently difficult to conciliate, a plausible explanation could be that either POA cells were missed by the mapping method employed in this study or the immunoreactivity of this population might be influenced by seasonality.

In seasonal breeders, kisspeptin cells are reported to undergo variations in their product synthesis as a consequence of photoperiodic changes that affect both ARC and POA cell populations in a species-specific fashion (10). In line with this, dromedary camels (displaying reproductive seasonality between November and April in Morocco) exhibit twice the number of immunoreactive kisspeptin cells in both the POA and ARC during the breeding season than in the non-breeding season, and this increase is more marked in females (40). Conversely, female llamas–and other South American camelids–are not considered seasonal breeders, exhibiting ovarian cyclicity, conception, and labour all year round (41–43) similarly as observed in the llama herd involved in the present study; in their natural habitat, breeding seasonality is associated to management practices, as a short wet and warm season restricts the abundance of high-quality forage between December and March in the high Andes (44). Whether llama kisspeptin cells exhibit seasonal variations remains to be determined.

In this study, quantification of GnRH and kisspeptin fibres in the ME revealed a higher prevalence of kisspeptin than of GnRH peptide at this level. Even though several studies (17, 45, 46) have investigated the morphological distribution of GnRH and kisspeptin fibres, no studies have described the concurrent densities of fibre innervation in the ME. In female rats, immunoelectron microscopy has revealed occasional direct synaptic contacts of kisspeptin-containing fibres to GnRH fibres; these animals presented rich innervation of both kisspeptin and GnRH fibres located in the internal and external zones of the ME, respectively (17). Furthermore, Pompolo et al. (47) reported colocalisation of GnRH and kisspeptin peptides in cells and fibres of the POA and also the ME in ewes. In the present study, colocalisation of these peptides was not detected in any of the brain regions analysed. Similarly as found here, variation in kisspeptin fibre distribution in the ME has been reported within single animals (46).

In several species (17, 45, 48), it has been described the presence of kisspeptin fibres in the ME, and even the release of measurable quantities of kisspeptin in the portal blood has been reported in the sheep (49). Furthermore, kisspeptin administration has been shown to stimulate GnRH release from the ME *in vitro* (17) and *in vivo* (50). In addition to this apparent effect within the ME boundaries, the rich kisspeptin innervation of the llama ME found in this study also suggests a potential hypophysiotropic role. Interestingly, Smith et al. (49) reported that *in vitro* kisspeptin application to pituitary gonadotroph cells (that express the kisspeptin receptor) during the follicular, but not luteal, phase prompts LH release in ewes.

GnRH cells appear to be a vital player in the ovulatory mechanism of llamas, as blockade of its peripheral receptors prevents LH-dependent ovulation (24). Since in New and Old World camelids ovulation occurs in response to the exogenous hormonal male β-NGF stimulus (23), attention has been given to the potential mechanism(s) involved in this response. Recently, kisspeptin has been proposed (25, 39) to be the mediator involved in the llama ovulatory mechanism under the assumption that exogenous kisspeptin effects mimic the actions of brain kisspeptin systems on GnRH cells (39), and yet this does not prove the physiological role of kisspeptins in the llama brain. It is unlikely that kisspeptins–including short forms (51, 52)–penetrate the blood–brain barrier in neuroactive amounts, and so it is thought (53) that, when given systemically, circulating kisspeptin acts locally on the OVLT, where rodent GnRH cells have been shown to extend their processes (54) similarly as reported here. Since oestradiol concentrations affect the magnitude of LH released following β-NGF administration in llamas (55), the rich kisspeptin innervation of the ME suggests that this is an area where oestrogen-sensitive kisspeptin cells might play a modulatory effect, for example, by affecting the amount of GnRH released in response to β-NGF. The physiological significance of the anatomical findings reported here for the ovulatory mechanism in llamas is still to be determined.

## Data Availability Statement

The datasets supporting the conclusions of the present study are available from the corresponding authors upon reasonable request. Requests to access the datasets should be directed to Luis Paiva, luis.paiva@postgrado.uach.cl; Marcelo Ratto, marceloratto@uach.cl.

## Ethics Statement

The animal study was reviewed and approved by the Bioethical Committee of the Universidad Austral de Chile, reference no. 253/2015.

## Author Contributions

MB and MR performed the animal experiments and tissue collection. LS, MB, and LP performed the immunohistochemistry. LP and LS analysed the images and data. LP wrote the manuscript. LP and MR edited and reviewed the manuscript. All authors contributed to the article and approved the submitted version.

## Conflict of Interest

The authors declare that the research was conducted in the absence of any commercial or financial relationships that could be construed as a potential conflict of interest.
